# Effect of Awareness of Excessive Use of Force on the Psychological Well-being and Workplace Environment of Emergency Physicians: A Pilot Study

**DOI:** 10.5811/westjem.48904

**Published:** 2026-05-18

**Authors:** Anisha Turner, Thomas Medrano, Kevin-Dat Nguyen, Xiaofan Huang, Richina Bicette, Vidya Eswaran, Adedoyin Adesina

**Affiliations:** * Baylor College of Medicine, Department of Emergency Medicine, Houston, Texas; † Baylor College of Medicine, Institute for Clinical and Translational Research, Houston, Texas

## Abstract

**Introduction:**

Excessive use of force by law enforcement officers is a critical public health issue linked to serious health consequences such as hypertension, post-traumatic stress disorder (PTSD), and depression. While emergency physicians (EP) are often the first to treat patients with excessive use of force-related injuries, the work-life and psychological toll of witnessing these incidents remains underexplored. In this study, we examine how awareness of and exposure to excessive use of force affects the psychological well-being and professional environment of EPs.

**Methods:**

An observational cross-sectional survey was developed by EPs and psychiatrists to assess work-life and psychological impacts of awareness of excessive use of force on EPs. The survey included multiple-choice and Likert-scale questions and used the Impact of Event Scale–Revised (IES-R). It was distributed anonymously to EPs at three Texas academic institutions. We used the Fisher exact test and the Wilcoxon rank-sum test to compare groups. Our primary outcome measure was psychological distress, assessed with the IES-R. Secondary outcome measures included self-reported effects of awareness of excessive use of force on subjects’ work environments, patient care, and interactions with law enforcement.

**Results:**

Of 282 surveys sent to EPs, 43 responded (15%). Eighteen of 40 (45%) reported experiencing work-life impacts and 15 of 40 (37.5%) experienced psychological distress; three did not comment. Abnormal IES-R scores were found in seven (19.6%) of 35 participants; eight did not respond. Participants who noted work-life effects of excessive use of force were more likely than those whose work-life was not affected to report modified patient care approaches (61% vs 0%, *P* < .001), altered interactions with law enforcement (83% vs 0%, *P* < .001), and altered interactions with patients (50% vs 0%, *P* < .001). Psychological distress was more prevalent among participants with personal exposure to excessive use of force compared to those without personal exposure (47% vs 12%, *P* = .02), and among those with second-hand exposure compared to those without second-hand exposure to excessive use of force (80% vs 56%, *P* = .04).

**Conclusion:**

This pilot study demonstrates that exposure to excessive use of force is associated with psychological distress and professional impact among emergency physicians, influencing interactions with patients and law enforcement. These findings underscore the need for further characterization of the effects of awareness of and exposure to excessive use of force on EPs. This, in turn, may inform institutional interventions and national protocols aimed at mitigating psychological burden, supporting physician resilience, and promoting high-quality, equitable patient care.

## INTRODUCTION

Excessive use of force by law enforcement officers, also referred to as police brutality, is defined as “excessive and unjustified use of force by a member of law enforcement.”^1^ While this term acknowledges the legitimate role of force in policing, it also recognizes the inappropriate application of force applied regardless of intentionality.^2^ Excessive use of force includes emotional and physical abuse, verbal assault, psychological intimidation, physical and sexual violence, and neglect.^3^ Prior studies have demonstrated that exposure to direct excessive use of force is associated with the development of stress-related chronic conditions such as diabetes, hypertension, and obesity, as well as mental health conditions like anxiety, depression, and post-traumatic stress.^4^

While the impact of excessive use of force on individual victims who directly interact with law enforcement officers is commonly appreciated, it extends beyond those immediate encounters. Secondary exposure—defined as exposure among individuals who do not directly experience force but are affected through witnessing, learning about, or anticipating such events—has also been associated with adverse physical and psychological outcomes.^5^ Studies demonstrate that observing or hearing about excessive use of force involving members of one’s community negatively affect collective health and well-being and is associated with elevated symptoms of psychological distress, even in the absence of direct interaction with law enforcement.^3^ Emerging evidence further suggests that vicarious exposure through social or familial networks or media coverage of excessive use of force incidents is also associated with stress-related health effects.^6^

Importantly, secondary exposure encompasses a spectrum of proximity, ranging from direct observation of excessive use of force in the same physical space to indirect exposure via shared narratives or media. Although physical proximity may intensify emotional and psychological responses, evidence suggests that even remote or mediated exposure can result in heightened vigilance and anticipatory stress related to potential future encounters with law enforcement officers.^7^ This heightened vigilance reflects a psychological preparedness for adverse interactions with law enforcement and underscores that secondary exposure to excessive use of force —regardless of physical proximity—can have meaningful health implications.

A group that may be disproportionally impacted by direct or secondhand exposure to excessive use of force is emergency physicians (EP). While no studies have confirmed a direct increase in exposure among EPs, they frequently work alongside law enforcement officers in the emergency department (ED) and routinely care for potential victims of excessive use of force-related encounters. Compared to most other medical specialties, EPs interact with law enforcement more often and occasionally witness excessive use of force during patient restraint.^8^ One study reported that 98% of surveyed EPs had treated patients with suspected excessive use of force.^9^ Another study reported that the presence of law enforcement officers in the ED negatively impacted patient care 10% of the time and affected clinicians 2% of the time.^10^ For physicians who share the same ethnicity as victims of excessive use of force, encounters have an indirect psychological toll. Some question how they might be treated outside their professional role, asking themselves, “how [would I be treated if I] didn’t have badges and scrubs.”^11^ Some EPs of color “even taped [their] hospital badges to [their] dashboards so [they] wouldn’t have to reach for them to prove who [they were]. [They had] seen what has happened when people of color reach for identification after being pulled over.”^11^

**Population Health Research Capsule**

What do we already know about this issue?

*Excessive use of force affects victims and communities, but its impact on the mental health and work life of emergency physicians (EP) is not well described*.

What was the research question?

*Is exposure to excessive use of force associated with psychological distress and work-life impact among EPs?*

What was the major finding of the study?

*Physicians reporting the impact of exposure to excessive use of force more often altered patient care (61% vs 0%, P < .001)*.

How does this improve population health?

*Identifying emergency physicians as an affected workforce can lead to trauma-informed interventions to protect their well-being and the quality of patient care*.

In this study, our objective was to examine the impact of excessive use of force on the psychological well-being and professional environment of EPs. We also explored whether these effects vary across different racial, ethnic, and sex groups, highlighting disparities in psychological distress and workplace dynamics.

## METHODS

### Study Design and Participants

We conducted an observational cross-sectional survey study to evaluate the psychosocial and professional impact of exposure to excessive use of force on EPs. The study protocol was reviewed by the Institutional Review Board at Baylor College of Medicine (H-48738), which approved an electronic consent process. The consent document was embedded at the beginning of the anonymous survey, and completion of the survey indicated participant consent. Eligible participants were practicing EPs in Texas who were fluent in English and self-reported awareness of excessive use of force. Non-physicians, non-emergency medicine specialists, and respondents who did not complete key outcome measures were excluded from analysis.

Points of contact at Texas academic centers that have EDs were asked to distribute an anonymous survey to department physicians, with a $20 electronic gift card offered as an incentive for participation. We received notification that the survey had been sent to the distribution list of three Texas academic institutions, composed of 282 physicians, between May–July 2023. Of the 43 emergency physicians who responded to the electronic survey, three were excluded due to incomplete data, and one was omitted.

### Survey Development

We developed the survey using a consensus-based, interdisciplinary approach. Emergency physicians contributed to the clinical context regarding intersections with law enforcement officers, ED workflows, and professional impacts of excessive use of force exposure. A psychiatrist provided expertise in trauma-related symptomatology and selection of validated psychological assessment tools. Together, the team identified domains related to psychological distress, work-life impact, patient care, and professional interactions for inclusion in the survey.

**Figure 1:**
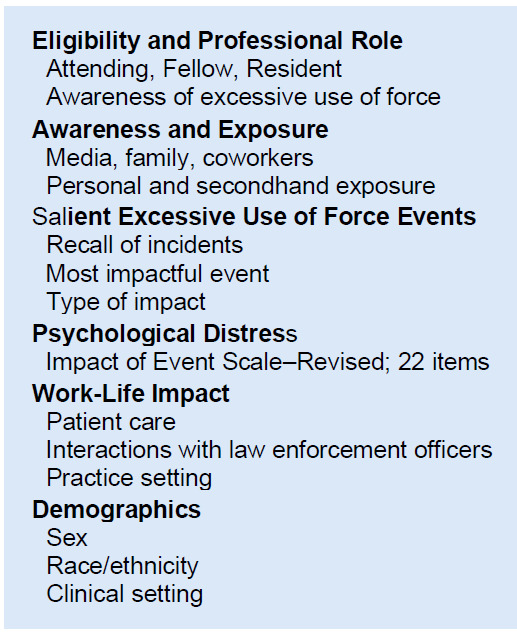
Structure and content domains of a survey on the impact of exposure to excessive use of force that was administered to emergency physicians.

Survey items were informed by a review of the existing literature on excessive use of force, vicarious trauma, and physician well-being. The final instrument included demographic questions, items assessing the work-related impact of excessive use of force using Likert scales, and the Impact of Event Scale-Revised (IES-R). The IES-R is a 22-question self-report scale designed to screen adults for psychological distress following a traumatic event. The overall structure and content domains of the survey instrument are illustrated in Figure 1.

### Impact of Event Scale

**Figure 2:**
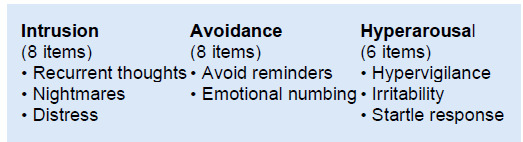
Domains of the Impact of Event Scale–Revised (IES-R), a 22-item self-report instrument used to assess trauma-related psychological distress. The scale comprises three subscales: intrusion, avoidance, and hyperarousal. Subscale scores are summed to generate a total IES-R score.

The IES-R is a 22-item, self-report scale designed to screen adults for psychological distress following a traumatic event. The scale has three subscales—intrusion (8 items), avoidance (8 items), and hyperarousal (6 items)—which together form the total score, as depicted in Figure 2. Participants rated how distressing each item had been over the prior seven days on a 5-point scale ranging from 0 (“not at all”) to 4 (“extremely”). The sub-scale scores were averaged, omitting any unanswered items, and the total scale was calculated by summing up the three sub-scale scores.

### Outcome Measures

Our primary outcome measure was psychological distress related to exposure to excessive use of force. Psychological distress was measured using the IES-R. Secondary outcomes included self-reported effects of excessive use of force on participants’ work environments, patient care, interactions with law enforcement officers, and preferred clinical practice settings.

### Work-Life Impact Measures

Participants reported the perceived impact of excessive use of force on their professional experiences using a 5-point Likert scale ranging from “strongly disagree that there is an impact” to “strongly agree.” Those who agreed or strongly agreed that exposure to excessive use of force impacted their work environments were prompted to specify the nature of the impact. Response options included increased medical errors, more near misses, altered interactions with patients, coworkers and law enforcement officers, changes to patient care approaches, and shifts in their preferred practice setting. Participants were also able to provide further details by selecting “other.”

### Procedures

The survey was administered using Research Electronic Data Capture (REDCap), a secure, web-based application designed to support data capture for research studies, hosted at Baylor College of Medicine.^12^,^13^ Prior to survey distribution, we pilot-tested the electronic questionnaire to ensure clarity, usability, and technical functionality. Survey responses were anonymous, and we collected demographic data (sex, race, professional role) without identifiers. To facilitate incentive distribution, participants who chose to receive the $20 electronic gift card provided an email address, which was stored separately from survey responses and not linked to study data.

### Data Analysis

We summarized participant responses using frequency with percentage or median with 25^th^ and 75^th^ percentiles and compared the responses between participants whose work life and mental health were/were not affected by exposure to excessive use of force. We analyzed categorical variables using the Fisher exact test, while continuous variables were assessed with the Wilcoxon rank-sum test. A significance level of 0.05 was used, and we performed all analyses using R statistical software v4.3.2 (R Foundation for Statistical Computing, Vienna, Austria).

## RESULTS

A total of 43 EPs responded to the survey. Three were omitted due to participants reporting they were not aware of the existence of excessive use of force. Of the respondents included in the study, 23 (53%) were attendings, 17 (40%) were residents, and three (7%) were fellows. Most respondents reported they had been made aware of excessive use of force through social media (79%, n = 34) or local news (77%, n = 33), while a quarter of respondents (23%, n = 10) reported that they had personal experience with excessive use of force. Fifteen participants (37.5%) reported being mentally impacted overall by incidents of excessive use of force, and 18 (45%) reported that incidents of excessive use of force had impacted their work life. The prevalence of psychological distress using IES-R was as follows: normal in 28 respondents (80%); mild in two respondents (5.7%); moderate in one respondent (2.9%); and severe in 11 respondents (11%) (Table 1).

### Psychological Impact

Participants who reported a mental impact from exposure to excessive use of force were more likely to have a personal experience with excessive use of force by law enforcement—either through direct encounters or incidents involving non-family members outside of work—compared to those who were not mentally impacted (47% vs 12%, *P* = .02; 40% vs 8%, *P* = .03) (Table 2). They rated their mental impact higher than their physical and emotional impact. Those psychologically distressed were more likely to avoid “[thinking]k about [instances of excessive use of force]” while at work compared to those who were not distressed (*P* = .04). Additionally, those who were mentally impacted reported that awareness of excessive use of force influenced their desired clinical practice setting, compared to those who were not mentally impacted (20% vs 0%, *P* = .05). Although nine participants (64%) in this group had normal IES-R scores, three (21.1%) had moderate to severe scores. This group also scored significantly higher in the avoidance sub-scale than those who were not mentally impacted (median 7 vs 3, *P* = .04).

Participants who reported psychological distress were more affected by the deaths of Breonna Taylor (33%, n = 5) and George Floyd (53%, n = 8) compared to those who were not psychologically impacted. (*P* = .04). Significant racial and ethnic differences emerged between mentally impacted and non-impacted participants; those affected were 8% Asian, 46% Black, and 46% White, whereas those not affected were 42% Asian, 17% Black, 13% Latinx, and 29% White.

### Work Environment Impact

Physicians whose work environment was affected by awareness of excessive use of force exhibited significant avoidance behaviors. They reported “[trying] not to think about it” (*P* = .04) or “[feeling] as if it hadn’t happened or wasn’t real” (*P* = .04) (Table 3). They also scored significantly higher on the intrusion sub-scale (median 6 vs 1, *P* = .05) Several notable changes in workplace interactions emerged among those impacted: they interacted differently with patients (50% vs 0%, *P* < .001) and with law enforcement officers (83% vs 0%, *P* < .001). Additionally, awareness of excessive use of force influenced the way they care for patients (61% vs 0%, *P* < .001).

While most of those with a work impact recognized race, ethnicity, and sex as risk factors for excessive use of force victimization, a higher proportion of physicians impacted by awareness of excessive use of force while at work identified religion as a risk factor compared to those without a work impact (28% vs 0%, *P* = .01). While most of the IES-R scores for this group were normal (65%, n = 11), 24% of this group had severe IES-R scores (n = 4).

### Differences in Race, Ethnicity and Sex

White respondents were significantly more likely than non-White respondents to alter their interactions with law enforcement officers following exposure to excessive use of force (*P* = .04). Black and Asian respondents were more likely to become aware of excessive use of force through family members (*P* < .01 and.04, respectively), highlighting differences in how information about these incidents is shared across racial groups. Additionally, Black respondents reported higher rates of physical symptoms associated with awareness of excessive use of force (*P* = .01). They also had significantly higher IES-R scores compared to non-Black respondents (*P* < .01). Female respondents were more likely to report emotional distress and symptoms related to exposure to excessive use of force (*P* = .02), suggesting a sex-based difference in psychological response. These findings emphasize the importance of considering racial and sex disparities when assessing the mental health and professional effects of awareness of excessive use of force on EPs.

## DISCUSSION

This study expands the current understanding of excessive use of force by law enforcement officers, demonstrating that its impact extends beyond patients and communities to affect EPs who routinely care for individuals involved in such encounters. While prior research has documented associations between negative encounters with law enforcement and adverse health outcomes among directly affected individuals,^3^,^4^ empirical studies examining how either direct or remote exposure to excessive use of force affects EPs themselves remain limited.

Our findings reveal that the impact of excessive use of force extends beyond those who have directly experienced it. Both direct and secondary exposure were associated with measurable psychological distress and meaningful changes in professional behavior among EPs, reinforcing the concept of excessive use of force as a broader occupational and public health concern. Notably, prior work by Hutson et al, while involving a larger national sample and a higher response rate, focused on EPs experiences with law enforcement presence in the emergency department and did not assess the psychological effects or professional consequences of exposure to excessive use of force. Our study extends this literature by directly examining trauma-related distress and professional impact associated with exposure among EPs.

### Excessive Use of Force as an Occupational Stressor for Emergency Physicians

While prior literature has focused primarily on the health consequences of excessive use of force for directly affected individuals and communities, our results highlight EPs as an under-recognized group experiencing secondary trauma related to exposure to excessive use of force. Over one-third of respondents reported psychological impacts, and nearly one-quarter screened positive for abnormal IES-R scores, suggesting clinically relevant distress. Such distress manifested in multiple ways. Those who reported psychological impacts were significantly more likely to engage in avoidance behaviors such as “trying not to think about it” or “talk about it.” Conversely, EPs who reported a work-related impact were more likely to experience intrusive thoughts such as “pictures [popping] into [their] heads.” These findings align with existing literature on secondary traumatic stress and vicarious trauma among healthcare workers who care for traumatized populations but extend this framework to excessive use of force as a specific and recurring source of exposure within emergency medicine.

Importantly, distress related to excessive use of force was not limited to internal psychological symptoms but translated into professional consequences. The EPs who reported work-life impact described altered patient care practices and changes in interactions with both patients and law enforcement. These findings add to the phenomenon known as the “cost of caring,” which has been studied by mental health therapists and law enforcement professionals.^14^,^15^ Research has shown that the “cost of caring” for traumatized individuals can lead to work-related stressors associated with secondary trauma such as burn out, vicarious trauma, secondary traumatic stress, and post-traumatic stress disorder. These findings raise concerns about how repeated exposure to excessive use of force may influence clinical decision-making, therapeutic relationships, and workplace dynamics in EDs, and emphasize the importance of further exploration in this area.

These findings also intersect with emerging literature on moral distress and moral injury among physicians, particularly when clinicians perceive a conflict between professional obligations to patient advocacy and the actions of external authorities. Exposure to excessive use of force—whether through direct clinical encounters or repeated secondary awareness—may place EPs in ethically fraught situations that challenge professional identity and moral frameworks. Conceptual support for this connection is reflected in a recent case analysis by Richards et al, which described moral injury and difficulty maintaining therapeutic trust among physicians treating patients subjected to excessive use of force during mental health crises.^16^ Although that analysis was not conducted among EPs and was limited to a case-based discussion within a legal-psychiatric context, it highlights mechanisms, such as erosion of trust, ethical conflict, and emotional distress, that are consistent with our findings. To our knowledge, this study is among the first to empirically examine the psychological and professional impact of exposure to excessive use of force among EPs through a moral injury and secondary trauma framework. Although moral injury was not directly measured, the observed patterns of avoidance, intrusive thoughts, and altered professional behaviors align with mechanisms described in moral injury and secondary traumatic stress literature.

### Racial and Ethnic Disparities in Excessive Use of Force-related Distress

Consistent with broader population-based studies, our findings demonstrate disproportionate psychological effects of excessive use of force among Black EPs, who had significantly higher IES-R scores compared to non-Black colleagues. This disparity mirrors prior research showing that awareness of police violence disproportionally affects the mental health of Black individuals, even in the absence of direct exposure. For example, one study reported that the awareness of events of excessive use of force through public channels disproportionately affects the mental health of people from racial and ethnic minorities, particularly Black and Latinx.^4^ Another study found that police killings of unarmed Black Americans contributed to over 50 million additional days of poor mental health per year among that demographic.^17^ Furthermore, members of the Black community who become aware of these incidents through social media and other sources experience a heightened sense of fear, apprehension, and distrust toward law enforcement.^17^ Given that Black physicians may simultaneously occupy the roles of healthcare professional and community member, exposure to excessive use of force may activate both professional and personal stress pathways.

Additionally, the intersection of racial identity and professional roles may compound distress. Although physicians may have socioeconomic protections relative to the general population, they are not insulated from the broader structural inequities and racializing policing practices affecting their communities. Studies reinforce that socioeconomic disparities further intensify the psychological toll of exposure to excessive use of force. Those in lower socioeconomic strata are more likely to experience excessive use of force, and Black Americans directly or secondarily impacted by excessive use of force, report living in fear due to awareness of these statistics and systemic injustices.^18^ The cumulative psychological burden described in the literature as “racial battle fatigue” may, therefore, extend into the clinical workspace, contributing to the heightened vigilance, emotional exhaustion, and distress among Black EPs.^19^ Such racial disparity further supports the framing of excessive use of force from a public health standpoint as a social determinant of health due to its broader impact on society.^14^

### Excessive Use of Force, Secondary Traumatic Stress, and Emergency Department Dynamics

Our findings support conceptualizing excessive use of force-related distress among EPs as a form of secondary traumatic stress, a form of post-traumatic stress disorder defined as “experiencing repeated or extreme exposure to aversive details of the traumatic event(s)” (*Diagnostic and Statistical Manual of Mental Disorders, 5th Ed*). Unlike many professionals, EPs occupy a unique position in that they often care for both victims of violence and individuals restrained by law enforcement, frequently in the presence of armed officers, which may contribute to re-traumatization or heightened secondary traumatic stress among clinicians.^20^ This proximity may heighten the risk of re-traumatization and exacerbate stress response, particularly when EPs are repeatedly exposed to encounters related to excessive use of force. When combined with physician burnout, an increasingly recognized issue, secondary traumatic stress can lead to compassion fatigue.^21^ Our data suggest that excessive use of force-related incidents are a critical component of this equation, especially among minoritized physicians who reported a disproportionately higher psychological burden in our study. Importantly, our findings indicate that secondary exposure, through media, colleagues, or community awareness, was associated with psychological distress similar to that observed with personal exposure, reinforcing that repeated indirect exposure alone may be sufficient to trigger clinically relevant stress responses among EPs.

The observed prevalence of abnormal IES-R scores in our cohort is comparable to previously reported rates of secondary traumatic stress among emergency clinicians. One study of EPs reported that 12–19% screened positive for secondary traumatic stress, and another found high rates among nurses in the ED.^22^, ^23^ Secondary traumatic stress has been linked to increased medical errors, reduced confidence, and absenteeism.^24^^,25^ Although our study did not directly assess these outcomes, the reported changes in patient care and professional interactions suggest that stress related to awareness of excessive use of force may contribute to similar downstream effects, with implications for both physician well-being and patient safety.

### Implications for Emergency Medicine Practice and Systems

To our knowledge, this study is among the first to empirically examine the association between personal and second-hand exposure to excessive use of force by law enforcement and trauma-related psychological distress and professional impact among EPs. Taken together, these findings suggest that excessive use of force should be recognized as an occupational stressor within emergency medicine, particularly for physicians from marginalized racial and ethnic groups. Institutions should consider targeted interventions to address excessive use of force-related distress, including structural debriefings following such encounters, access to trauma-informed mental health support, and education on secondary traumatic stress and coping strategies.

At the systems level, EDs may benefit from interdisciplinary collaboration between clinicians, hospital leadership, mental health professionals, and law enforcement agencies to establish protocols that minimize re-traumatization while preserving necessary working relationships. Incorporating excessive use of force-related stress into broader physician wellness and burnout prevention initiatives may further enhance institutional support.

These findings underscore the importance of recognizing excessive use of force as an occupational stressor for emergency physicians and the need for healthcare leaders to acknowledge and address its effects. Physicians and healthcare institutions may benefit from collaborative efforts to develop accessible, non-stigmatized mental health resources and trauma-informed support systems for clinicians exposed to excessive use of force-related encounters both within and outside the workplace. Interdisciplinary collaboration among clinicians, hospital leadership, mental health professionals, and law enforcement partners may help mitigate secondary traumatization while preserving effective working relationships in the ED. Although our study did not evaluate specific institutional or policy interventions, these findings suggest that examining the role and impact of law enforcement presence in the ED, as well as policies that influence clinician exposure to such encounters, may be important areas for future inquiry. Framing excessive use of force within a broader public health context—as a potential social determinant of health—may facilitate more comprehensive approaches to addressing its impact on patients, communities, and the healthcare professionals who care for them.

### Future Directions

Future research should aim to better characterize the longitudinal impact of excessive use of force exposure on EPs, including its relationship to burnout, retention, clinical outcomes, and patient trust. Larger, multi-institutional studies are needed to confirm these findings and explore protective factors that may mitigate excessive use of force-related distress. Additionally, qualitative studies may help elucidate the lived experiences of EPs navigating such exposure and identify institution-specific opportunities for intervention,

By recognizing EPs as stakeholders and an at-risk population affected by excessive use of force, this study contributes to a more comprehensive understanding of excessive use of force as a public health issue and underscores the importance of supporting the mental health and professional well-being of clinicians working at the intersection of healthcare and law enforcement. Acknowledging the toll of this exposure underscores the need for targeted interventions to address this form of trauma and mitigate its effects on EPs. It is important that healthcare administration remains mindful of the various ways this trauma may manifest among EPs, ensuring adequate support mechanisms are in place. Additionally, we recommend that institutions consider a collaborative and interdisciplinary approach to address these challenges while preserving the working relationship between EPs and law enforcement officers.

## LIMITATIONS

This was a hypothesis-generating study, which is the first to assess the impact of excessive use of force on EPs. This study had a small sample size and sampling bias based on voluntary participation. All respondents worked clinically at Texas academic centers, which may have resulted in different exposures to law enforcement officers or victims compared to other clinical sites. The survey asked respondents potentially sensitive questions regarding their mental health, and it is possible that social desirability bias influenced responses. Because the events of excessive use of force referenced in the survey occurred more than a year before the survey was distributed, recall bias may have also played a role in responses. Furthermore, physician baseline levels of mental distress or PTSD were unknown, and there may be unmeasured confounders that affect levels of mental distress due to excessive use of force. Given these limitations, larger and multi-site studies are essential to better understand the role of excessive use of force on EPs. Because the survey did not explicitly assess moral injury or longitudinal outcomes, future studies should directly evaluate these constructs to better characterize the ethical and professional consequences of exposure among EPs.

## CONCLUSION

The findings from our study indicate that exposure to excessive use of force by law enforcement officers is associated with meaningful psychological distress and professional impact among emergency physicians. Our data suggests that this exposure—both direct and secondary—is linked to changes in workplace experience, patient care interactions, and mental well-being. Additionally, EPs from racial and ethnic minority groups are disproportionately affected, experiencing an inherent psychological impact, which highlights potential disparities in how excessive use of force-related stress is experienced within the emergency medicine workforce.

## Figures and Tables

**Table 1:** Characteristics of emergency physicians who responded to a survey regarding how exposure to excessive use of force affected them mentally and professionally, with a subset showing signs of psychological distress.

Characteristic	N	N=40^1^
Employment category	40	
Attending		21/40 (53%)
Fellow		3/40 (7.5%)
Resident		16/40 (40%)
Sex	39	
Male		20/39 (51%)
Female		19/39 (49%)
Ethnicity	37	
White		13/37 (35%)
Black		10/37 (27%)
Hispanic/Latinx		3/37 (8.1%)
Asian		11/37 (30%)
By what means were you made aware of excessive use of force		
Local radio stations	40	19/40 (48%)
Social media	40	34/40 (85%)
Local news	40	33/40 (83%)
Family members	40	10/40 (25%)
Co-workers	40	16/40 (40%)
Personal experience	40	10/40 (25%)
Within the past 24 months, my mental health has been impacted by knowing about incidences of police excessive use of force	40	
Agree		15/40 (38%)
Disagree/Neutral		25/40 (63%)
Within the past 24 months, knowing about incidents of police brutality and violence has impacted you while at work.	40	
Agree		18/40 (45%)
Disagree/Neutral		22/40 (55%)
Impact of Event Scale Score	35	
Normal		28/35 (80%)
Mild		2/35 (5.7%)
Moderate		1/35 (2.9%)
Severe		4/35 (11%)
IES sub-categories		
Hyperarousal	35	1 (0.00, 3.00)
Avoidance	35	4 (1.50, 9.50)
Intrusion	35	2 (0.00, 7.50)
N reported the total number of non-missing observations		

1 Median (Q1, Q3); n/N (%)

**Table 2:** Comparison of the psychological/mental impact on emergency physicians of exposure to excessive use of force, with those who were affected likely to have had personal experiences with law enforcement, exhibit avoidance behaviors, and to report influence on their preferred clinical practice setting.

Characteristic	N	Overall^1^ (N = 40)	Agree^1^ (n = 15)	Disagree/neutral^1^ (n = 25)	*P* value^2^
Race/Ethnicity	37				.04
White		13 / 37 (35%)	6/13(46%)	7/24(29%)	
Black		10 / 37 (27%)	6/13(46%)	4/24(17%)	
Hispanic/Latinx		3 / 37 (8.1%)	0/13(0%)	3/24(13%)	
Asian		11 / 37 (30%)	1/13(7.7%)	10/24(42%)	
Made aware of excessive use of force by law enforcement through one of the following:					
Personal experience*	40	16 / 40 (40%)	7/15 (47%)	3/25 (12%)	.02
Co-workers	40	16 / 40 (40%)	7/15 (47%)	9/25 (36%)	.50
Local news	40	33 / 40 (83%)	13/15 (87%)	20/25 (80%)	.70
Social media	40	34 / 40 (85%)	12/15 (80%)	22/25 (88%)	.70
Local radio	40	19 / 40 (48%)	7/15 (47%)	12/25 (48%)	> .90
How did awareness of excessive use of force impact you?					
Mental/Psychologically	40	28 / 40 (70%)	14/15 (93%)	14/25 (56%)	.02
Emotionally	40	26 / 40 (65%)	12/15 (80%)	14/25 (56%)	.20
Physically	40	5 / 40 (13%)	3/15 (20%)	2/25 (8%)	.30
In what ways have you personally experienced excessive use of force by law enforcement?					
I have not personally experienced or witnessed police brutality	40	17 / 40 (43%)	3/15 (20%)	14/25 (56%)	.05
Police brutality toward co-workers	40	1 / 40 (2.5%)	0/15 (0%)	1/25 (4.0%)	> .90
Police brutality toward myself	40	2 / 40 (5.0%)	1/15 (6.7%)	1/25 (4.0%)	> .90
Police brutality toward family members	40	1 / 40 (2.5%)	1/15 (6.7%)	0/25 (0%)	.40
Police brutality toward non-family members outside work	40	8 / 40 (20%)	6/15(40%)	2/25 (8.0%)	.04
Any reminder brought back feelings about it	35				.04
Not at all		17/35 (49%)	5/14 (36%)	12/21 (57%)	
A little bit		9/35 (26%)	2/14 (14%)	7/21 (33%)	
Moderately		7/35 (20%)	6/14 (43%)	1/21 (4.8%)	
Quite a bit		2/35 (5.7%)	1/14 (7.1%)	1/21 (4.8%)	
Extremely		0/35 (0%)	0/14 (0%)	0/21 (0%)	
(Missing)		5	1	4	
IES sub-categories					
Intrusion	35	2.00(0.00,7.50)	4.50(0.25,10.75)	2.00(0.00,6.00)	.50
Hyperarousal	35	1.00(0.00,3.00)	2.00(0.00,3.00)	0.00(0.00,2.00)	.08
Avoidance	35	4.00(1.50,9.50)	7.00(3.25,16.25)	3.00(0.00,6.00)	.04
How has knowing about incidents of police excessive use of force impacted you while at work					
Affected my desired practice setting	40	3 / 40 (7.5%)	3/15 (20%)	0/25 (0%)	.05
Increased near misses	40	1 / 40 (2.5%)	1/15 (6.7%)	0/25 (0%)	.40
Interact differently with patients	40	9 / 40 (23%)	3/15 (20%)	6/25 (24%)	>.90
Interact differently with co-workers	40	3 / 40 (7.5%)	1/15 (6.7%)	2/25 (8%)	>.90
Interact differently with police officers	40	15 / 40 (38%)	7/15 (47%)	8/25 (32%)	.50
Affected the way I care for patients	40	11 / 40 (28%)	6/15 (40%)	5/25 (20%)	.30

1 n/N (%).

2 Fisher exact test; Wilcoxon rank-sum test.

*IES*, Impact of Event Scale.

**Table 3:** Emergency physician who reported work-related effects of awareness of excessive use of force demonstrated greater psychological intrusion and avoidance, and reported changes in patient care, law enforcement interactions, and clinical behavior.

Characteristic	N	Overall^1^ (N = 40)	Agree^1^ (n = 18)	Disagree/neutral^1^ (n = 22)	*P* value^2^
IES Total Score	35				.04
Abnormal		7 / 35 (20%)	6/17 (35%)	1/18 (5.6%)	
Normal		28 / 35 (80%)	11/17 (65%)	17/18 (94%)	
IES Score					.06
Normal		28 / 35 (80%)	11/17 (65%)	17/18 (94%)	
Mild		2 / 35 (5.7%)	1/17 (5.9%)	1/18 (5.6%)	
Moderate		1 / 35 (2.9%)	1/17 (5.9%)	0/18 (0%)	
Severe		4 / 35 (11%)	4/17 (24%)	0/18 (0%)	
IES Sub-Categories					
Hyperarousal		1.00(0.00,3.00)	2.00(0.00,4.00)	0.00(0.00,1.00)	.06
Avoidance		4.00(1.50,9.50)	6.00(2.00,14.00)	3.00(1.25,5.75)	.3
Intrusion		2.00(0.00,7.50)	6.00(0.00,11.00)	1.00(0.00,4.25)	.05
IES Scale: “How much have you been distressed or bothered by these difficulties?” (significant findings)					
I avoided letting myself get upset when I thought about it or was reminded of it.	33	12 / 33 (36%)	9/16 (56%)	3/17 (18%)	.02
I felt as if it hadn’t happened or wasn’t real.	35	4 / 35 (11%)	4/17 (24%)	0/18 (0%)	.05
I tried not to think about it.	32	5 / 32 (16%)	5/16 (31%)	0/16 (0%)	.04
How has knowing about incidents of police excessive use of force impacted you while at work?					
Affected the way I care for patients	40	11 / 40 (28%)	11/18 (61%)	0/22 (0%)	<.001
Interact differently with police officers	40	15 / 40 (38%)	15/18 (83%)	0/22 (0%)	<.001
Interact differently with patients	40	9 / 40 (23%)	9/18 (50%)	0/22 (0%)	<.001
Interact differently with co-workers	40	3 / 40 (7.5%)	3/18(17%)	0/22(0%)	.08
Affected my desired practice setting	40	3 / 40 (7.5%)	3/18(17%)	0/22(0%)	.08
Increased medical errors	40	0 / 40 (0%)	0/18(0%)	0/22(0%)	>.90
Increased near misses	40	1 / 40 (2.5%)	1/18(5.6%)	0/22(0%)	.50
What characteristic(s) of victims of police brutality contribute to excessive use of force by law enforcement?					
Religion	40	5 / 40 (13%)	5/18 (28%)	0/22 (0%)	.01
Race/ethnicity	40	40 / 40 (100%)	18/18 (100%)	21/22 (95%)	>.90
Sex	39	19 / 39 (49%)	15/18 (83%)	14/22 (64%)	.30
Socioeconomic status	40	30/40 (75%)	5/18 (83%)	15/22 (68%)	.50
Mental illness	40	37/40 (93%)	16/18 (89%)	21/22 (95%)	.60
Substance use	40	34/40 (85%)	16/18 (89%)	18/22 (82%)	.70

1 n/N (%).

2 Fisher exact test; Wilcoxon rank-sum test.

*IES*, Impact of Event Scale.
